# JOSEPH T. FREEMAN AWARD AND EXCELLENCE IN REHABILITATION OF AGING PERSONS AWARD PRESENTATIONS AND LECTURES

**DOI:** 10.1093/geroni/igae098.0937

**Published:** 2024-12-31

**Authors:** Kirsten Corazzini

**Affiliations:** Chair

## Abstract

The Joseph T. Freeman Award lecture will feature an address by the 2024 Freeman Award recipient Ali Ahmed, MD, FGSA of the Washing DC VA Medical Center and the 2024 Excellence in Rehabilitation of Aging Persons Award recipient Roger A. Fielding, PhD, FGSA of Tufts University. The Joseph T. Freeman Award is a lectureship in geriatrics awarded to a prominent clinician in the field of aging, both in research and practice. The award was established in 1977 through a bequest from a patient’s estate as a tribute to Dr. Joseph T. Freeman. The Excellence in Rehabilitation of Aging Persons Award is designed to acknowledge outstanding contributions in the field of the rehabilitation of aging individuals.

